# Promoting physical activity among adolescent girls: the *Girls in Sport* group randomized trial

**DOI:** 10.1186/s12966-017-0535-6

**Published:** 2017-06-21

**Authors:** Anthony D. Okely, David R. Lubans, Philip J. Morgan, Wayne Cotton, Louisa Peralta, Judith Miller, Marijka Batterham, Xanne Janssen

**Affiliations:** 1 Early Start Research Institute and School of Education, University of Wollongong, Wollongong, NSW 2522 Australia; 2 llawarra Health and Medical Research Institute, Wollongong, NSW 2522 Australia; 3 Priority Research Centre in Physical Activity and Nutrition, School of Education, University of Newcastle, Newcastle, 2308 Australia; 4 Faculty of Education and Social Work, The University of Sydney, Sydney, NSW 2006 Australia; 5 School of Education, University of New England, Armidale, NSW 2351 Australia; 6 National Institute for Applied Statistics Research Australia, University of Wollongong, Wollongong, NSW 2522 Australia; 7 University of Strathclyde, School of Psychological Sciences and Health, Glasgow, Scotland

**Keywords:** Physical activity, Youth, School, Randomized controlled trial, Health promoting schools, Rural

## Abstract

**Background:**

Slowing the decline in participation in physical activity among adolescent girls is a public health priority. This study reports the outcomes from a multi-component school-based intervention (*Girls in Sport*), focused on promoting physical activity among adolescent girls.

**Methods:**

Group randomized controlled trial in 24 secondary schools (12 intervention and 12 control). Assessments were conducted at baseline (2009) and at 18 months post-baseline (2010). The setting was secondary schools in urban, regional and rural areas of New South Wales, Australia. All girls in Grade 8 in 2009 who attended these schools were invited to participate in the study (*N* = 1769). Using a Health Promoting Schools and Action Learning Frameworks, each school formed a committee and developed an action plan for promoting physical activity among Grade 8 girls. The action plan incorporated strategies in three main areas – i) the formal curriculum, ii) school environment, and iii) home/school/community links – based on the results of formative data from target girls and staff and on individual needs of the school. A member of the research team supported each school throughout the intervention. The main outcome measure was accelerometer-derived total physical activity (TPA) spent in physical activity. Data were analyzed from December 2011 to March 2012.

**Results:**

1518 girls (mean age 13.6y ±0.02) were assessed at baseline. There was a significant decline in TPA from baseline to 18-month follow-up with no differences between girls in the intervention and control schools. Only one-third of schools (4/12) implemented the intervention as per their action plan. Per-protocol analyses on these schools revealed a smaller decline in percentage of time spent in MVPA among girls in the intervention group (adjusted difference 0.5%, 95% CI = -0.01, 0.99, *P* = 0.05).

**Conclusions:**

The *Girls in Sport* intervention was not effective in reducing the decline in physical activity among adolescent girls. Lack of implementation by most intervention schools was the main reason for a null effect. Identifying strategies to enhance implementation levels is critical to determining the true potential of this intervention approach.

**Trial registration:**

This study was retrospectively registered with the Australian New Zealand Clinical Trials Registry ACTRN12610001077055.

Date of registration: 7 December 2010.

**Electronic supplementary material:**

The online version of this article (doi:10.1186/s12966-017-0535-6) contains supplementary material, which is available to authorized users.

## Background

Recent global data show that the prevalence of recommended levels of physical activity are less than 20% among adolescent girls [1]. Moreover, these levels decline precipitously into adulthood [2, 3], highlighting the importance of intervening during the adolescence period. Evidence from systematic reviews [4, 5] show that the most effective school-based interventions among adolescents used whole-of-school approaches that link curricula activities with the broader school environment and local community.

Recent reviews on the effectiveness of school-based interventions among adolescent females have found only small effects when physical activity was measured objectively [6–8]. These authors recommend more studies that include an objective measure of physical activity, with follow-ups longer than 12 months, and that are guided by formative research with the target population.

The *Girls in Sport* intervention was part of an initiative in the state of New South Wales (NSW), Australia called the Premier’s Sporting Challenge [9]. This Challenge aimed to promote participation in sport and physical activity among children and young people attending government schools (approx. 70% of students). As part of the Challenge, the NSW Department of Education and Communities (DEC) commissioned a consortium of researchers to work with the Department’s School Sport Unit and selected school communities to design, implement, and evaluate a multi-component school-based initiative to promote physical activity among adolescent girls. *Girls in Sport* incorporated components of previously successful school-based interventions among adolescent girls [10–12] such as enhanced school sport, changes to the school ethos and strengthening community-based links, along with extensive formative research, to create school and community environments that promoted physical activity among adolescent girls through school sport, physical education, recreation, and leisure time activities. The primary aim of *Girls in Sport* was to determine if an 18-month school-based intervention targeting school sport, the school environment, and links with the local community could slow the decline in physical activity among adolescent girls compared with matched control schools that did not receive the intervention.

## Methods

### Study design and setting

This was a group randomized controlled trial with outcomes assessed at baseline and 18-months post-randomization follow-up. It involved 24 secondary schools across seven regions in the state of New South Wales, Australia. There were no variations to the methods after the trial had commenced. The detailed methods of the study have been previously published and are readily available through an open access journal [13].

### School and participant selection and recruitment

The NSW DEC sought expressions of interest from school principals (*N* = 500). Recruitment and consent of schools occurred in October 2008. Within each school, all girls in Grade 8 in 2009 were invited to participate. Girls needed to provide written consent from themselves and their parents. If a student or parent did not consent, they still participated in the intervention but did not in data collection.

### Randomization

The school was the unit of randomization. Schools were matched into 12 pairs based on the following criteria: school size, proportion of students from non-English speaking and Indigenous backgrounds, teaching experience of PE staff, organization of school sport, school type, and geographic location. Each matched pair of school was randomly allocated, using a computer-based random number producing algorithm to intervention or control group by a researcher independent of the project who then communicated to the research team who informed each school of its allocation. Due to the timeframe of the study, formative research was required to be conducted in the intervention schools in October/November, 2008. This meant randomization needed to occur prior to baseline data collection.

### Formative research

The formative research identified the needs and interests of adolescent girls, and from school staff, their perceptions of community facilitators and barriers to girls’ participation in physical activity. Interviews were held with relevant staff (including PE and non-PE teaching staff and a member of the school executive), and separate focus groups were conducted with groups of boys, and with girls in Grade 8. The Project Officer from the NSW DEC (SM) attended along with the Project Manager (LMP), who collected the data. In addition, participants were asked to map community physical activity facilities and opportunities on provided maps of the school and local community. Informal observations of PE lessons, recess and lunchtime activities were also conducted by the Project Manager. These were conducted in each of the 12 intervention schools. Between 1 and 3 observations were made during recess, lunch, and in PE lessons where possible and these were recorded as field notes.

Results suggested two main reasons schools were interested in participating in the study. These were the chance to “revitalize” sport in their school and the opportunity to engage particular groups of girls who were currently not participating enthusiastically in PE or sport. Staff believed the reasons for girls not participating in school sport were: 1) how it was structured, which lacked variety and limited choice, with those who were less skilled and confident being the last to choose a sport; 2) the lack of resources; and 3) the lack of expertise among non-PE teaching staff who supervised school sport. Among the girls, the main reasons for their non-participation were similar to those reported by staff, with additional barriers identified including the “dominating” behaviors of boys during PE and sport and their perceived lack of skills and confidence.

Girls were asked what they would like included in a school sport program. They suggested the opportunity to choose some of the activities (especially non-traditional activities) and to participate with their friends; having motivated teachers, more modern sports uniforms, and more respectful behavior from boys; and higher levels of activity during sessions. These suggestions were consistent with those cited in the literature [12, 14–16].

Results were provided to each school and school committees were asked to consider them when developing their intervention strategies and action plans. For example, staff were encouraged to survey girls to determine what activities they would like to participate in and then examine ways some of them could be integrated into their school sport programs. Schools were advised of the importance of students being a part of the school sports committee so they had a “voice” in their school.

### Intervention

Using a Health Promoting Schools framework [17, 18] and Action Learning approach [19], schools developed unique 18-month action plans. A member of the research team was assigned to be a “critical friend” at each school. This involved working with the school as it collected and interpreted its own data and assisting with the development and implementation of the school’s action plan [20]. The intervention strategies were designed to achieve the primary aim of the project. This aim was the same for each school, to prevent a decline in participation in moderate- to vigorous-intensity physical activity (MVPA) levels among girls over the course of the intervention. Secondary outcomes for the project were for schools to provide programs better designed to meet the needs of girls and to make girls aware of these, and more functional links to activities in the community. These were measured through the process evaluation. Improving confidence and self-efficacy in physical activity participation was also an additional outcome that was assessed through a psyschosocial questionnaire. Each school followed an identical process in developing their intervention. This involved:

1. Forming an action learning team (referred to as a Committee) within their school community. Schools were advised that this committee should comprise an executive level teacher, program champion, at least two other teachers, and at least two female students from the designated Grade group.
2. Developing school-specific action plans in three areas based on the results of the formative research and on individual needs of the school.

These three areas that constituted the “active component” of the intervention were the “formal” curriculum, school environment, and links with the community [21]. A specific description of how schools typically targeted each of these areas is described as follows:

#### Formal curriculum

The main focus of this area was to enhance school sport. Schools did this by trying to engage girls for at least 50% of the class time in moderate- to vigorous-intensity physical activity (MVPA) while reducing time spent in management tasks (e.g., organising students). An additional aim was for teachers to promote physical activity in and out of class. Activities were also targeted that girls indicated through the formative research that they would like to participate in. These included recreational and non-traditional activities such as power walking, yoga, Pilates, Zumba®, boxing-related fitness, and skipping activities.

#### School environment

Modifying the school ethos or environment aimed to raise awareness of the benefits of physical activity and sport at the school level. Schools took the approach that making sustainable changes involved more than implementing activities – it required a change in how they valued school sport and physical activity and embedded it into the school culture. Each school’s action learning team (or Committee) drove this intervention component. The Committee’s role was to advocate for the intervention within the school. They were responsible for developing and implementing initiatives that they believed would be sustainable. Schools were provided with information from the research team to inform the development of this component [10, 13, 22–25], which included lunchtime and after-school activity programs, modifying school policies related to use of equipment and facilities, allowing students to wear sports uniforms to school on sports days, gala afternoons, and aligning the school Awards/Merit system with the intervention.

#### Links with the community

To enhance links with the local community, schools sought professional development from the research team in how to promote out of school activity during the school sport sessions. This included making explicit links, such as using local facilities (e.g., fitness centres, PCYC’s, indoor rock climbing and sports centres). Others involved teachers identifying and prompting sports and activities available in the local community (e.g., basketball, touch football, tennis, soccer) during school sport and organising representatives (such as Development Officers) from these organisations to visit the school. Local community providers were also asked to offer classes and incentives for girls to participate.

Each school’s specific action plan formed part of the overall school plan for the year. School plans provide a framework to drive change within a school over a 3-year period in areas such as student engagement and retention, teacher quality, and connected learning [26]. Schools documented strategies to address each of the outcomes of their action plan, including who would be involved and how they would measure success.

During the intervention, schools participated in monthly meetings with their critical friend to share their progress towards the study outcomes. Schools were encouraged to modify strategies and, if further assistance was required, this was discussed between the research team and NSW DEC staff and communicated back to the school.

Support was given to the schools through funding from the NSW DEC to support implementation and for staff to attend professional development activities. These activities included an initial two-day training program, and a two-day research symposium mid-way through the intervention period (Feb 2010). In addition, if a school requested specific professional development in an area and a member of the research team was able to provide this, they did so. For example, several of the schools wanted suggestions for activities to run in a lunch-time gym class/boot camp for girls and one of the research team delivered a professional development session for them on this. Regular contact was provided by the *Girls in Sport* Project Manager who was employed by the NSW DEC.

### Control schools

Control schools continued with their usual programs. At the conclusion of the project these schools received training and materials related to the findings of the project. Staff also attended the final Research Colloquium in February 2011.

### Data collection procedures

Trained data collectors were blinded to group allocation. Baseline data were collected between February, 2009 and June, 2009 and follow-up data between July, 2010 and December, 2010. Data were collected at the same time in each pair of matched schools. Teachers and students were kept blinded to their matched comparison school. Each data collector was given a detailed manual, checklist and scripts to read when informing the participants of the measures.

### Measures

#### Primary outcome

Accelerometer measured total physical activity (TPA) [27] spent in physical activity was the primary outcome for the study. All participants wore an Actigraph accelerometer (7164 and GT1M models; Fort Walton Beach, FL) for one week. Thirty-second activity counts were uploaded to determine time spent in light (1.5 to 3.9 METs) moderate (4.0 to 6.9) and vigorous (≥7.0) activity. Age-specific count ranges relating to the above intensity levels were based on prediction equations for energy expenditure [28]. Values were calculated for percentage of monitored time spent in light, moderate, and vigorous physical activity to account for variation in wear time. Participants also received two text messages reminding them to wear the accelerometers and to return them at the end of the 7-day period.

#### Secondary outcomes

Psychosocial outcomes were assessed by questionnaire and included enjoyment of physical activity and school sport [29], physical activity self-efficacy [30], peer support for physical activity [31], social support during school sport [32], strategies to increase physical activity [33], school physical activity environment, physical self-concept, and perceived importance of physical activity [34]. Validity and reliability testing of all psychosocial outcomes has been reported [13].

#### Process evaluation

At the end of the first year, schools documented their progress towards the study outcomes based on implementation of their specific strategies. Interviews were conducted at the end of the intervention with each *Girls in Sport* school committee, staff, and students to assess the extent to which the strategies were implemented. Girls were asked to indicate if there had been any changes to school sport over the past 18 months, if they were asked to suggest how to improve school sport, if they were informed about sports in the local community, and if any lunchtime activity programs were implemented.

#### Qualitative data

There was a qualitative component to this project. It was designed to triangulate the quantitative results and provide knowledge about how *Girls in Sport* was implemented in each school, what influenced what happened in each school and whether the project had an impact on the girls targeted by the school. That is, the girls who seemed to be most disengaged from school sport and physical activity. The qualitative component, through its descriptions of what seemed to be the most and least successful components of the project and schools responses to the project, was also able to provide some recommendations and ideas for future policy and practice in relation to school based sport and physical activity outside the PE lesson.

The qualitative data were collected through the following methods:

- Formative individual and group interviews were conducted prior to the implementation of the project in each intervention school with the executive, both PE teachers and teachers from other key learning areas, at least eight Year 8 girls and three or four Year 8 boys at most schools;
- Individual and group interviews in comparison schools;
- Identification of the most disengaged case study girls (six per school): one individual interview and one final group interview;
- Final individual and group interviews with executive, teachers, girls and some boys where possible in the intervention and comparison schools;
- Ongoing observations by the critical friend on school visits, school reports at workshops and *Girls in Sport* conferences, school planning documents and regular Chief Investigator reports on the progress of the school with which they were associated.

These data are not reported on in this study but are being written up separately. However, qualitative data have been used to explain some of the quantitative findings in this paper.

### Sample size

In the absence of a reliable estimate of the intra-class correlation for the primary outcome measure of accelerometer-derived total physical activity (TPA) an estimate of 0.01 was used in the *a priori* calculations. Effect sizes and variance estimates, 77.51 (SD102.92) TPA, which was 18.4% of the baseline mean, were obtained from a previous study [10]. Using these figures, a model based on a critical *t-*value of 2.228 (taking into consideration the matching of the schools) was obtained for estimates based on 12 schools per group. Variance estimates were adjusted for clustering as proposed by Murray [35] where the standard error of the estimate in the usual *t*-estimation was replaced by $\sqrt{\frac{2\left({\widehat{\sigma}}_m^2+ m{\widehat{\sigma}}_g^2\right.}{ m g}}$ where ${\widehat{\sigma}}_m^2$ is the estimate of the unadjusted subject component of the variance, ${\widehat{\sigma}}_g^2$ is the unadjusted school component of the variance, *m* is the number of subjects per school and *g* is the number of schools per group. Sample sizes as low as 10 participants per school completing the study provided adequate power (>80% power and *P* < 0.05). Given that the estimate of effect could be considered optimistic for the present design a more modest effect size (10% of baseline mean, 42.07 TPA) was also considered. It was also anticipated that group sizes would vary between schools and therefore the estimates were based on a harmonic mean of 30 participants per school completing [36]. With this conservative mean effect size and a harmonic mean sample size of 30 completing the study, the power still remained high (0.987).

### Statistical analyses

Statistical analyses of the primary outcome variable, accelerometer-derived counts per minute, were performed using a linear mixed model (PROC MIXED) in SAS (version 9.2, SAS Inc, Cary NC) between December 2011 and March 2012. All models accounted for the hierarchical structure of the data. Analyses were not adjusted for multiple comparisons. Analyses involving mins/day for light, moderate, vigorous, and MVPA were adjusted for accelerometer type. Student data were included in the analyses if the accelerometer was worn for >600mins/day on at least 3 days [37]. Analyses followed intention-to-treat principles. An advantage of the linear mixed model is that it can incorporate all available data allowing for the analysis of partial datasets created when a participant drops out of the study or misses a study visit. Imputation of missing data was also performed on the accelerometer data due to the large amount of missing data at follow-up. This imputation was performed using PROC MI and MIANALYSE. Sensitivity analyses were performed. Linear mixed models were also used to analyze all continuous accelerometer-derived outcome variables (time in minutes and percentage of time spent in sedentary behavior, light, moderate and vigorous intensity physical activity) and all secondary outcome variables.

Per protocol analyses were also completed on four schools that were deemed, *a priori* to have met all the criteria for having implemented the intervention as planned. These criteria included six key areas (see Additional file 1: Table S1). These were compared with their matched control school. During the analyses, one of the schools and its matched control school were too small to include in the analysis. As such, only three intervention schools were analyzed together and compared with their matching schools.

## Results

**Fig. 1:**
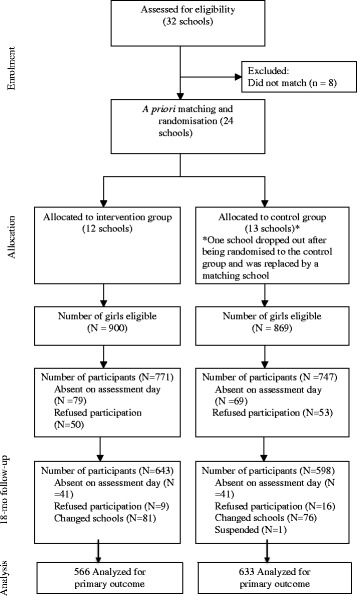
Flow of participants through the study

Thirty-two schools from seven geographical regions expressed interest in participating in the study and were assessed for eligibility. Eight of these were unable to be matched, leaving 24 that were pair-matched and randomly allocated. One school withdrew after being allocated to the control group and was replaced with another school that was nominated by the NSW DEC as a suitable matching school. The flow of schools and students through the study are displayed in Fig. 1. Eighty-six percent of eligible girls completed baseline assessments.

Useable accelerometry data was obtained from almost 80% of girls at baseline (*n* = 1199) and 43% at follow-up (*n* = 653). The main reason for the low amount of useable data at follow-up was girls being absent on the day of testing and when they were subsequently approached on return to school, refused to wear the accelerometer. Baseline characteristics of the sample have been reported previously [13] and there were no differences between groups on any of the variables. There was no difference between groups in the retention rate of participants (83% vs 80%, respectively) and no differences between girls who dropped out of the study compared with those who were retained with respect to age (*P* = 0.33). Compared with girls who dropped out, girls who completed the study had a significantly lower level of TPA (*P* = 0.01) and of time and percentage of time spent in MVPA (both *P* = 0.02). The mean age of the girls was 13.6 y (±0.02). The sample demonstrated low levels of physical activity participation. Only 1.5% met the current Australian guidelines of ≥60 mins of MVPA every day [38].

### Primary outcome analysis

Table 1 reports the results for the intention-to-treat analyses for physical activity and sedentary behavior outcomes. There was a significant decline among girls in both the intervention and control schools for all physical activity outcomes from baseline to follow-up, and a significant increase in sedentary behavior. There were no differences in the changes from baseline to follow-up between girls in the intervention and control schools.

**Table 1:** Intention-to-treat analysis for primary outcomes (accelerometer-derived physical activity)

	Baseline		Follow-up		Est. of effect (95% CI)	Time	*P* values	
	Intervention	Control	Intervention	Control			Group	Interaction
	(*n* = 566)	(*n* = 633)	(*n* = 306)	(*n* = 298)				
Total PA (TPA), median (IQR^b^)	407.23 (337.26,498.31)	401.39 (323.47,492.16)	358.60 (300.45,467.37)	358.00 (287.54,447.79)	2.16 (-40.79, 45.11)	<0.0001	0.3825	0.8944
						0.0001	0.5058	0.6782^a^
Sedentary activity, mins/day (95% CI)	476.10 (430.08,521.11)	480.43 (432.46,534.95)	497.43 (454.58,543.88)	508.2 (464.70,558.29)	-2.81 (-21.74, 16.19)	<0.0001	0.3953	0.8667
Sedentary activity, %time/day (95% CI)	60.39 (55.36,65.51)	61.48 (56.49,66.53)	65.25 (59.60,69.84)	65.62 (60.22,69.91)	0.25 (-1.58, 2.07)	<0.0001	0.3155	0.7362
Light activity, mins/day (95% CI)	271.50 (238.85,308.21)	261.87 (226.28,296.54)	239.61 (206.30,283.15)	239.14 (203.30,278.57)	-4.69 (-17.10, 7.73)	<0.0001	0.4776	0.5100
Light activity, %time/day (95% CI)	34.69 (30.43,39.03)	33.71 (29.46,38.23)	32.30 (27.59,37.43)	31.36 (26.57,35.72)	0.34 (-0.91, 1.59)	<0.0001	0.2841	0.5543
Moderate activity, mins/day (95% CI)	33.13 (24.39,43.35)	32.23 (23.50,42.27)	25.65 (16.90,38.70)	25.00 (17.36,34.75)	-0.53 (-5.91, 4.85)	<0.0001	0.1997	0.8390
Moderate activity, %time/day (95% CI)	4.30 (3.10,5.57)	4.09 (2.97,5.43)	3.37 (2.15,4.98)	3.16 (2.18,4.43)	-0.02 (-0.76, 0.71)	<0.0001	0.1443	0.8898
Vigorous activity, mins/day (95% CI)	2.40 (1.08,4.82)	2.82 (1.17,5.35)	1.33 (0.48,2.90)	1.50 (0.50,3.50)	0.24 (-0.93, 1.41)	<0.0001	0.5136	0.7126
Vigorous activity, %time/day (95% CI)	0.33 (0.14,0.63)	0.35 (0.15,0.69)	0.17 (0.06,0.37)	0.19 (0.06,0.44)	0.02 (-0.12, 0.16)	<0.0001	0.5108	0.8482
Moderate-to-vigorous activity, mins/day (95% CI)	36.21 (26.27,47.33)	35.29 (25.41,47.02)	27.13 (17.71,42.31)	27.00 (18.25,37.83)	-0.35 (-6.58, 5.87)	<0.0001	0.3372	0.8481
						<0.0001	0.3871	0.7608^a^
Moderate-to-vigorous activity, %time/day (95% CI)	4.67 (3.39,6.19)	4.53 (3.20,6.14)	3.58 (2.29,5.24)	3.47 (2.36,4.81)	-0.04 (-0.87, 0.79)	<0.0001	0.2669	0.8500
						<0.0001	0.3169	0.7763^a^

Linear mixed models; ^a^ *P* values represent those combined from 10 imputed datasets, calculated using PROC MI in SAS; ^b^ *IQR* interquartile range

### Per-protocol analysis

Table 2 reports the results for the per protocol analyses. There were no differences between groups for any of the physical activity outcomes. One of the variables (percentage of time spent in MVPA/day) did show a difference that was almost statistically significant (*P* = 0.05) in favor of the intervention group. Girls in the intervention schools had a 0.5% smaller decline in MVPA than girls in the control schools.

**Table 2:** Per protocol analysis for accelerometry-derived physical activity outcomes

	Baseline		Follow-up		Estimate of effect (95%CI)	Time	*P* values	
	Intervention	Control	Intervention	Control			Group	Interaction
	(*N* = 196)	(*N* = 208)	(*N* = 121)	(*N* = 122)				
Total PA (TPA), mean (SD)^a^	385.09 (122.21)	361.42 (106.74)	376.47 (120.91)	343.02 (101.76)	13.70 (-24.47, 51.87)	0.1396	0.2975	0.3757
Sedentary activity, %time/day (95% CI)	62.84 (61.81,63.87)	64.14 (63.20,65.08)	66.43 (65.14,67.71)	67.70 (66.54,68.87)	-0.29 (-1.90, 1.32)	0.0002	0.6435	0.7923
Light activity, %time/day (95% CI)	32.81 (31.92,33.70)	31.66 (30.85,32.48)	29.93 (28.77,31.08)	29.17 (28.06,30.28)	-0.11 (-1.57, 1.34)	0.0003	0.8462	0.9786
Moderate activity, %time/day (95% CI)	4.01 (3.76,4.26)	3.83 (3.62,4.04)	3.77 (3.43,4.10)	3.20 (2.95,3.45)	0.44 (-0.02, 0.90)	0.0077	0.2049	0.0890
Vigorous activity, %time/day (95% CI)	0.34 (0.29,0.40)	0.37 (0.31,0.42)	0.26 (0.19,0.33)	0.25 (0.19,0.31)	-0.002 (-0.20, 0.20)	0.0071	0.7856	0.6466
Moderate-to-vigorous activity, %time/day (95% CI)	4.35 (4.07,4.63)	4.20 (3.95,4.45)	4.01 (3.64,4.38)	3.45 (3.18,3.71)	0.49 (-0.005, 0.99)	<0.001	0.030 0.05	0.05

Results from linear mixed models; ^a^ *SD* Standard deviation

### Secondary outcome analysis

Table 3 reports the results for the psychosocial outcomes. There were significant declines from baseline to follow-up for all the outcomes except perceptions of physical conditioning (average decline for all variables across both groups = -0.17 units). The decline was significantly smaller in the intervention group (-0.14) than the control group (-0.24) for physical activity self-efficacy and was significantly greater in the intervention group (-0.26) than the control group (-0.11) for peer support for physical activity.

**Table 3:** Results from intention-to-treat analyses for psychosocial outcomes (questionnaire derived). All values Mean (SD) unless otherwise specified

	Baseline		Follow-up		Adjusted Diff (95%CI)	Time	*P* values	
	Intervention	Control	Intervention	Control			Group	Interaction
	(*n* = 767)	(*n* = 745)	(*n* = 636)	(*n* = 605)				
Self-perceptions Physical self-worth	2.67 (0.66)	2.74 (0.64)	2.52 (0.59)	2.56 (0.63)	0.03 (-0.05, 0.11)	<0.0001	0.269	0.430
Sports competency	2.57 (0.63)	2.62 (0.60)	2.49 (0.56)	2.50 (0.61)	0.05 (-0.02, 0.12)	<0.0001	0.496	0.138
Physical conditioning	2.67 (0.62)	2.73 (0.60)	2.56 (0.58)	2.56 (0.60)	0.06 (-0.04, 0.16)	0.1077	0.370	0.206
Body attractiveness	2.34 (0.66)	2.40 (0.66)	2.23 (0.62)	2.30 (0.67)	0.06 (-0.04, 0.16)	<0.0001	0.564	0.247
Physical strength	2.52 (0.57)	2.56 (0.54)	2.46 (0.52)	2.47 (0.56)	-0.02 (-0.11, 0.07)	<0.0001	0.098	0.587
Perceived importance of physical activity	2.80 (0.53)	2.80 (0.50)	2.73 (0.48)	2.75 (0.49)	-0.01 (-0.09, 0.06)	<0.0001	0.998	0.742
Physical activity self- efficacy	3.72 (0.55)	3.73 (0.59)	3.58 (0.62)	3.49 (0.67)	0.09 (0.003, 0.18)	<0.0001	0.687	0.043
Enjoyment of physical activity	4.25 (0.64)	4.23 (0.64)	4.02 (0.75)	4.00 (0.79)	0.01 (-0.10, 0.13)	<0.0001	0.711	0.806
Enjoyment of school sport	4.01 (0.75)	3.91 (0.76)	3.66 (0.85)	3.57 (0.87)	0.01 (-0.18, 0.20)	<0.0001	0.077	0.945
Social support for physical activity	3.14 (0.72)	3.01 (0.74)	2.88 (0.75)	2.90 (0.77)	-0.16 (-0.31, -0.01)	0.0001	0.530	0.038
Social support during school sport	3.97 (0.73)	3.94 (0.70)	3.72 (0.81)	3.77 (0.79)	-0.04 (-0.23, 0.15)	0.0001	0.780	0.643
Identification of strategies to increase physical activity	3.62 (0.71)	3.56 (0.75)	3.38 (0.79)	3.36 (0.80)	-0.04 (-0.14, 0.07)	<0.0001	0.601	0.482
Perceptions of school activity environment	3.75 (0.62)	3.70 (0.61)	3.61 (0.65)	3.54 (0.66)	0.01 (-0.12. 0.13)	<0.0001	0.721	0.914

Table 4 reports the physical activity results just for the school day. There were no differences between groups for any of the accelerometer-based outcomes.

**Table 4:** Intention-to-treat analyses for school-day physical activity

	Baseline^a^		Follow-up^a^		Estimate of effect (95%CI)	Time	*P* values	
	Intervention	Control	Intervention	Control			Group	Interaction
	(*n* = 630)	(*n* = 601)	(*n* = 416)	(*n* = 332)				
Total PA (TPA), Mean (SD)	96.85 (38.93)	98.43 (47.50)	96.83 (44.38)	93.74 (37.12)	1.49 (-13.64, 16.64)	0.6060	0.7445	0.9668
Percentage time sedentary activity, Mean (SD)	63.44 (8.38)	63.53 (9.05)	67.28 (7.59)	67.69 (7.02)	-0.76 (-3.62, 2.10)	<0.0001	0.8819	0.5375
Percentage time light activity, Mean (SD)	31.69 (7.35)	31.36 (7.77)	28.64 (6.67)	28.68 (6.34)	0.21 (-1.77, 2.20)	<0.0001	0.7681	0.9706
Percentage time moderate activity, Mean (SD)	4.52 (2.21)	4.62 (2.47)	3.80 (2.23)	3.37 (1.70)	0.34 (-0.84, 1.52)	0.0002	0.8054	0.5746
Percentage time vigorous activity, Mean (SD)	0.35 (0.46)	0.50 (0.87)	0.29 (0.50)	0.26 (0.39)	0.13 (-0.09, 0.35)	0.0019	0.5580	0.2237
Percentage time moderate-to-vigorous activity, Mean (SD)	4.87 (2.48)	5.12 (2.83)	4.09 (2.48)	3.63 (1.88)	0.46 (-0.93, 1.84)	<0.0001	0.8863	0.5329

Linear mixed models; ^a^unadjusted means and SD

### Process evaluation findings

Implementation data revealed that only 4 of the 12 schools implemented the intervention as intended. Further, nearly half the schools (5/12) met less than 50% of the implementation criteria and one-quarter of the schools met less than 25% of the criteria. Additional file 2: Table S2 reports how girls perceived the changes that were meant to occur as a result of their school implementing their *Girls in Sport* action plan. Compared with girls in the control schools, girls in the intervention schools were more aware of a female-specific initiative to promote physical activity in their school and noticed changes in school sport. Students in the intervention schools also did not notice any changes in school policies around greater access for girls or in being provided with information about sports and physical activities they could access and participate in outside of school hours. There were no reported adverse effects in the intervention schools as a result of the study.

## Discussion

The *Girls in Sport* intervention had no effect on reducing the decline in physical activity; however, findings from the per-protocol analyses were more encouraging and did show a small, and close to significant effect for percentage of time spent in MVPA. The smaller decline in MVPA among girls in the intervention schools equated to approximately 2.5mins/day. As these adolescent girls spent less than 5% of their day in MVPA and less than 2% met physical activity recommendations every day, this difference may be important.

There were several reasons why the intervention was not implemented as intended in many of the schools, which was the main reason for the null findings for the intention-to-treat analyses. First, schools found it difficult to overcome barriers such as resistance to changing the school culture among some staff, changes in staff who were on the school committee or who were the program champions, and low commitment levels from some school executives. Although implementation data showed that girls noticed schools were making an effort to address their needs, these efforts were not enough to make a significant change in their physical activity participation. Girls in the intervention schools reported changes in the types of activities on offer, the amount of time devoted to each activity, and the ways in which sports activities were chosen. Perhaps why these were not translated into changes in physical activity was because they did not happen consistently enough and only occurred in a very small number of schools (those included in the per protocol analyses). In addition, qualitative data suggested that the changes that resulted from the girls’ feedback were often in the form of “one-off” activities that involved most of the girls in the intervention schools. While girls found these enjoyable, they did not result in sustainable, better-designed programs to meet their needs [39].

Second, many schools found it challenging to sustain the initial momentum of change. Girls preferred activities that ran for a shorter duration and were changed more frequently (perhaps at the end of each term – every 10 weeks). However, teachers reported that to do this would require additional resources. Most schools responded to girls’ requests to have separate girls-only sport and PE classes. Again, qualitative data from girls reported that this enhanced their desire to participate in sport and PE; however, this did not result in changes in physical activity across the school day. This may have been due to other factors such as the training of staff and their level of supervision of school sport or the low intensity of many of the activities schools decided to target. It is recommended that teachers divulge greater responsibility to the students for such activities [40].

A further factor contributing to the null finding that came through in the qualitative data was an inability of schools to respond during the intervention period to suggestions made by girls in the formative research (see Methods section). The most notable of these was girls identifying that a reason for not participating in sport was the sports uniforms. Girls felt their current uniforms were not modern, made from uncomfortable translucent fabric and made them look and feel like boys. Despite girls on the sports committees recommending more modern uniforms, process data showed that this was not supported by schools in their interventions. This has been previously identified as a barrier to teenage girls participating in PE and school sport [12].

Schools identified through the qualitative data that adequately training all staff who would be supervising sport was key to successful implementation of this project. In government high schools in NSW, sport is usually structured so that all staff are timetabled and allocated workload to supervise a sport. This works well when students are supervised by teachers who are interested in, and who have the skills to supervise school sport in a way that fosters a motivating environment for the students. A major challenge is when staff are unmotivated and not adequately trained. Intervention schools sought to overcome this by providing professional development for all staff but this was not well attended, was not a large enough ‘dose’ (only a ‘one-off’ session ranging from 1-5 hours), and was rarely sustained beyond the initial session. Intervention schools then sought to only assign teachers who were motivated and trained to the classes that involved girls in the intervention. However, this was not always possible.

Results from the process evaluation showed that the intervention was not successful in increasing the links between the school and the local community with no differences between intervention and control schools in how informed girls were about these facilities and their impact on their participation outside of school hours (Additional file 2: Table S2). This may be due to many girls being already quite involved in physical activity or sport outside of school. In some areas, transport was a problem and for many of the girls other commitments were prioritized over sport.

The process evaluation also showed that all schools had the functional links with community sports and the facilities necessary for running a sports afternoon. However, as schools explored further options as part of this study they often expanded these links – for example to involve local gyms. In only a small number of schools were opportunities to more actively link female students with community sports taken up beyond notices in newsletters.

Results from the per protocol analyses were more encouraging and show that, when the intervention was implemented as intended, the decline in physical activity among adolescent females was reduced to almost statistically significant levels. A pleasing aspect about these results is that they occurred across a range of school types, strengthening the external validity of the intervention approach. Identifying the factors that enhanced the level of implementation in these schools is important to guide future interventions. Process data collected suggest that these schools had one or more of the following components: 1) an enthusiastic, passionate Program Champion; 2) strategies to ensure the sustainability of the project; 3) active support from the school principal; and 4) a functioning school committee. The importance of providing adequate support for the in-school program champion cannot be underestimated in addressing the issue of poor implementation. It has been identified as a key factor in successful school-based physical activity interventions among adolescents [40, 41].

Consistent with other studies in this age group [42], there was a decline in all psychosocial outcomes over the course of the intervention (Table 3). There was, however, an effect on physical activity self-efficacy. These results are consistent with other school-based physical activity interventions among adolescent girls that have found positive effects on self-efficacy but no subsequent effect on physical activity participation. Consistent with Social Cognitive Theory and Harter’s model of self-esteem [43], there are several factors that influence physical self-esteem that were targeted in this intervention. These include greater social support from teachers and providing activities and experiences that were enjoyable and sought to enhance girls’ perceived competence. That girls felt they were consulted about ways to improve school sport, and they felt that these suggestions were used as an indication of greater social support from teachers.

Females in the intervention schools reported a significantly greater decline in peer support for physical activity compared with their control schools counterparts (Table 3). As this instrument only assessed peer support, it is possible that the greater decline seen in the intervention group was due to heightened awareness and more perceived support for physical activity being provided by teachers. As a result, girls felt that they were less reliant on social support from their peers.

### Limitations

Strengths of the study include the cluster RCT design, objective measurement of physical activity – from around 80% of females at baseline, robust measures of secondary psychosocial outcomes, and the extensive formative research that guided the development of the intervention approach. Limitations include the low amount of useable accelerometry data at follow-up, with less than half of the females wearing the monitor for the required amount of time. As such, a large amount of data needed to be imputed. The likely impact of this was that the study may have been underpowered at follow-up and the imputations may have resulted in biased estimates, however given that the imputations demonstrated no effect of the intervention this is not likely in this case. This limitation is unfortunately common in interventions that use accelerometers among adolescents [44]. A further limitation was that none of the analyses adjusted for multiple comparisons.

## Conclusions

The *Girls in Sport* intervention was not effective in reducing the decline in physical activity among adolescent females. This lack of effectiveness was largely due to intervention schools not implementing the intervention as intended. This study reinforces that multi-component interventions are challenging to implement. In those schools that did demonstrate high levels of implementation, there was a smaller decline in the percentage of time spent in MVPA. Further research is needed to examine how to enhance and maintain implementation levels in diverse secondary school settings, against the backdrop of real-world challenges such as key staff leaving the school, lack of succession planning, and changing school culture in an area perceived as unimportant by many staff. Developing interventions that school staff will be motivated to implement for the duration of the intervention period and that the females involved are motivated to support are urgently needed. This study shows that it is possible to prevent a decline in MVPA among adolescent girls if schools fully implement the action plans they develop. The challenge for schools and researchers is how to work together in a collegial and beneficial way to maximize the likelihood of high levels of implementation in school-based interventions.

## Additional files

Additional file 1: Table S1. Girls in Sport Criteria Table. (DOCX 20 kb)

Additional file 2: Table S2. Perceptions of girls regarding changes that occurred in their school as a result of *Girls in Sport.* Responses from girls in the intervention schools were compared with those from the control schools (*N* = 1241). (DOCX 111 kb)
