# Supplementary material for: Promoting physical activity among adolescent girls: the Girls in Sport group randomized trial
Source: Int J Behav Nutr Phys Act. 2017 Jun 21;14:81. doi: 10.1186/s12966-017-0535-6 (PMC5480114; doi:10.1186/s12966-017-0535-6)
Supplement: Supplementary file 1 — Girls in Sport Criteria Table. (DOCX 20 kb) [file 12966_2017_535_MOESM1_ESM.docx]

| **Girls in Sport Criteria Table** |  |  |  |  |  |  |  |  |  |  |  |  |
| --- | --- | --- | --- | --- | --- | --- | --- | --- | --- | --- | --- | --- |
| **1. A School Committee that had all the following elements:** | | | | | | | | | | | | |
| - comprised at least one HT or DP |  |  |  |  |  |  |  |  |  |  |  |  |
| - included at least one teacher from another faculty (not PDHPE) |  |  |  |  |  |  |  |  |  |  |  |  |
| - Showed a willingness to consult students representative of the profile of the project (e.g., Year 8 girls, SRC, inactive girls etc) |  |  |  |  |  |  |  |  |  |  |  |  |
| - Was functional (defined as meeting regularly [at least 3 times] over the study period) |  |  |  |  |  |  |  |  |  |  |  |  |
| **2. Submitted a school action plan that had all the following:** | | | | | | | | | | | | |
| - was submitted on time |  |  |  |  |  |  |  |  |  |  |  |  |
| - attempted to address all key areas (whole school approach) with realistic and feasible goals that were reflective of what was identified in the formative data and covered/emphasised at the workshops and retreats (linked to GIS Objectives). |  |  |  |  |  |  |  |  |  |  |  |  |
| - evidence of consultation with their critical friend |  |  |  |  |  |  |  |  |  |  |  |  |
| - evidence of appropriate planning for sustainable change |  |  |  |  |  |  |  |  |  |  |  |  |
| **3. Made observable changes to the structure and delivery of school sport that were sustained for the duration of the intervention (observable to their critical friend and to Lauren/Sue).**For example, offered a single-sex option and/or provided a modified choice and then evaluated this. |  |  |  |  |  |  |  |  |  |  |  |  |
| **4. Evidence of sustainable changes made to at least one aspect of the school environment** (e.g., lunchtime programs). Sustainable should be defined here as these changes continued until the end of the intervention period and into this year (2011**).** |  |  |  |  |  |  |  |  |  |  |  |  |
| **5. Demonstrated a link to local infrastructure (resources and facilities) for the participating girls** (e.g., school provided information about local resources and facilities and incentives to use them that went beyond simply bringing outside groups into the school with no link back to tangible opportunities for the girls to participate in these activities in the community) |  |  |  |  |  |  |  |  |  |  |  |  |
| **6. Conducted TPL that involved at least one initiatives (such as TPL run by the critical friend or DEC; internal TPL run by School Committee for other staff)** |  |  |  |  |  |  |  |  |  |  |  |  |
