# Supplementary material for: Promoting physical activity among adolescent girls: the Girls in Sport group randomized trial
Source: Int J Behav Nutr Phys Act. 2017 Jun 21;14:81. doi: 10.1186/s12966-017-0535-6 (PMC5480114; doi:10.1186/s12966-017-0535-6)
Supplement: Supplementary file 2 — Perceptions of girls regarding changes that occurred in their school as a result of Girls in Sport. Responses from girls in the intervention schools were compared with those from the control schools (N = 1241). (DOCX 111 kb) [file 12966_2017_535_MOESM2_ESM.docx]

## Supplementary Table 2. Perceptions of girls regarding changes that occurred in their school as a result of *Girls in Sport.* Responses from girls in the intervention schools were compared with those from the control schools (N=1241)

| At my school over the past two years… | Effect estimate | P Value | Odds Ratioa | 95% CI |
| --- | --- | --- | --- | --- |
|  | (SEM) |  |  |  |
| I was aware there was a *Girls in* | 2.56 (0.57) | 0.0002 | 12.98 | 3.91, 43.06 |
| *Sport* project  I have noticed changes in school | 1.19 (0.30) | 0.0008 | 3.30 | 1.76, 6.19 |
| sport  My participation in school sport has | 0.33 (0.18) | 0.07 | 1.39 | 0.97, 2.01 |
| increased  If my participation in school sport | 0.93 (0.23) | 0.0006 | 2.54 | 1.58, 4.09 |
| has increased this was due to changes in school sport |  |  |  |  |
| I was asked to provide suggestions on how to improve school sport | 1.78 (0.36) | <0.0001 | 5.96 | 2.83, 12.55 |
| If I was asked to provide suggestions on how to improve | 1.40 (0.43) | 0.004 | 4.06 | 1.66, 9.91 |
| school sport, there was evidence that my ideas were used |  |  |  |  |
| I was provided with information about community sports and | 0.21 (0.26) | 0.44 | 1.23 | 0.72, 2.11 |
| activities that I could access and participate in outside of school. |  |  |  |  |
| If I was provided with such information, as a result of this I have | 0.42 (0.26) | 0.12 | 1.52 | 0.89, 2.59 |
| participated in some of these community sports and activities |  |  |  |  |
| Lunchtime physical activity programs for girls were | 0.98 (0.64) | 0.14 | 2.66 | 0.70, 10.08 |
| implemented.  If lunchtime activity programs were | 0.71 (0.45) | 0.13 | 2.02 | 0.80, 5.14 |
| implemented, I participated in them There were girls-only playground | 0.10 (0.72) | 0.89 | 1.10 | 0.25, 4.93 |
| areas and equipment  If there were girls-only areas and | 0.38 (0.58) | 0.52 | 1.46 | 0.44, 4.88 |

equipment then I used them

aLikelihood of the intervention group giving a ‘Yes” response to the question compared with the control group. A higher odds ratio indicates a greater likelihood among the intervention group. If the confidence intervals (95% CI) do not cross ‘1’, then this likelihood is statistically significant. E.g., for Q1, girls in the intervention group were nearly 13 times (12.98) more likely to be aware of the GIS project in their school than girls in the control group and this increased likelihood was statistically significant.
